# Organic–Inorganic Substitution Improves Cucumber Yield and Quality by Enhancing Carbon and Nitrogen Metabolism in Cucumbers

**DOI:** 10.3390/plants15142157

**Published:** 2026-07-13

**Authors:** Bingyan Li, Rui Liu, Xiukuan Jin, Hui Wang, Mingyue Li, Wei Gao, Di Wu

**Affiliations:** 1 Institute of Agricultural Resources and Environment, Tianjin Academy of Agricultural Sciences, Tianjin 300381, China; 2 Institute of Agro-Product Safety and Nutrition, Tianjin Academy of Agricultural Sciences, Tianjin 300381, China; 3 Cotton Research Institute of Shanxi Agricultural University, Yuncheng 044000, China

**Keywords:** organic–inorganic substitution, yield, quality, carbon–nitrogen metabolism, antioxidant level, cucumber

## Abstract

Replacing chemical fertilizers with organic fertilizers can improve soil quality and continuously increase crop yields, but its impact on cucumber growth remains unclear. This study, based on a seven-year field trial, explored the effects of five different fertilization strategies: no fertilization, application of inorganic fertilizer (NPK), 2/3 NPK + 1/3 organic fertilizer (M), 1/3 NPK + 2/3 M, and M on cucumber carbon and nitrogen metabolism, antioxidant levels, yield, and quality. The results showed that both NPK and M applications were beneficial to improving cucumber yield and quality. However, NPK alone led to the accumulation of malondialdehyde (MDA) in leaves, while NPK + M could improve its total antioxidant capacity. Furthermore, 1/3 NPK + 2/3 M significantly promoted the accumulation of amino acids and glucose in leaves and increased the activities of glutamine synthase, sucrose phosphate synthase, and other enzymes. Further analysis revealed that the content of glucose and protein in leaves was closely related to the synthesis of soluble sugars and vitamin C in fruits, with sucrose and MDA showing the most significant effects from different fertilization strategies. In conclusion, the application of 1/3 NPK + 2/3 M significantly improved the carbon and nitrogen metabolism activity of cucumbers, enhanced their stress resistance, and promoted yield and quality.

## 1. Introduction

Cucumbers are rich in polyphenols, carotenoids, and various vitamins and are considered one of the most nutritious and healthy vegetables [1]. As one of the most widely grown vegetables in China, the planting area of cucumber accounts for 56% of the world’s cucumber planting area, and its output accounts for more than 80% of the world’s total cucumber production [2]. Due to the high economic value of cucumbers, agricultural production systems are accustomed to increasing the availability of soil nutrients by applying large amounts of inorganic fertilizers to further increase cucumber yields. However, this practice has led to a series of serious environmental problems, such as soil salinization, groundwater pollution, and greenhouse gas emissions [3].

Sustainable vegetable cultivation requires the rational management of nutrient resources, maintaining soil fertility and reducing negative environmental effects. Manure is rich in nutrients and is often used as an organic fertilizer to increase crop yields and improve soil structure and microecological environment [4]. At the same time, using manure in farmland also helps reduce its environmental damage and disposal costs. The livestock industry produces more than 4 billion manures each year, but only a small part is applied to farmland [5,6]. Given that the application of manure is a cost-effective and environmentally friendly strategy, it is of great significance to improve soil fertility and crop productivity. Therefore, organic fertilizers such as manure are considered to be a better alternative to inorganic fertilizers [7]. The nutrient content of organic fertilizers is relatively low, and the release of nutrients is relatively slow. In contrast, inorganic fertilizers contain all the essential nutrients that plants can directly absorb. During the critical stages of plant growth and development, inorganic fertilizers are more commonly used rather than organic fertilizers [8]. Recent studies have found that, compared with the application of inorganic or organic fertilizers alone, replacing part of the chemical fertilizer with organic fertilizer (OFS) can significantly improve the plant height, leaf area, fruit fresh weight, and post-harvest quality of cucumbers, while also improving soil nutrient status and physical properties [9,10].

Plants obtain various forms of nitrogen from the soil, including ammonium nitrogen, nitrate nitrogen, amide nitrogen, etc., through processes such as absorption, translocation, and assimilation [11]. When nitrogen is sufficient, plants mainly produce high-nitrogen compounds, while when nitrogen supply is insufficient, metabolism will shift more to carbon-containing compounds and non-nitrogen-containing secondary metabolites. These substances help to improve the plant’s stress resistance to a certain extent, thereby maintaining the plant’s survival under limited nutrient supply [12]. Since the relative difference in nutrient release from organic and inorganic fertilizers leads to changes in soil nitrogen availability, and the impact of OFS on crop growth under multi-year continuous cropping patterns is still unclear [13], we hypothesize that OFS may affect the carbon and nitrogen metabolic cycle activities of the aboveground parts of plants. Based on a seven-year field experiment, we explored the changes in the cucumber’s antioxidant system, yield, quality, carbon metabolism, and nitrogen assimilation activities and their interactions, providing guidance for improving crop yield through OFS.

## 2. Materials and Methods

### 2.1. Experimental Sites

The experiment started in May 2018 at the Tianjin Academy of Agricultural Sciences (116°56′ E, 39°25′ N). The region has an average annual temperature of 14.1 °C, an average annual sunshine of 2752 h, and an average annual rainfall of 589.5 mm, with most rainfall concentrated in July, August, and September (Table S1 supplements the total monthly rainfall and average temperature during the cucumber growing season from 2018 to 2024). The soil type is fluvo-aquic soil. Before the experiment, the organic matter, total nitrogen, available phosphorus, and available potassium were 18.53, 0.77, 0.02, and 0.30 g/kg, respectively, and the pH was 8.3 in the surface soil (0–20 cm).

### 2.2. Experimental Design

This experiment adopted a randomized block design and included 5 treatments: no fertilizer (CK), all inorganic fertilizer (NPK), 2/3 inorganic fertilizer plus 1/3 organic fertilizer (2/3 NPK + 1/3 M), 1/3 inorganic fertilizer plus 2/3 organic fertilizer (1/3 NPK + 2/3 M), and all organic fertilizer (M). Each treatment contained 3 replications, a total of 15 plots, and each plot had an area of 36 m^2^ (6 m long × 6 m wide). Since the start of the experiment in 2018, the same management model was used for cucumber production (planting after the cucumber growing season, removing the plants from the experimental plots, and allowing the land to fallow until the start of the following year, with regular manual weeding during this period). The tested cucumber variety was Jinyou 409 (bred by the Tianjin Kerun Agricultural Technology Co., Ltd., Tianjin, China). Before planting, the soil was tilled, sun-dried, and furrowed. The cucumbers were planted on May 1st, with a plant spacing of 40 cm and a row spacing of 75 cm. After planting, the cucumbers were covered with mulch. The field layout and fertilization information are shown in Figure S1 and Table 1. Except for the CK treatment, the total nutrient input was the same for all other fertilization treatments. The application rates of nitrogen, phosphorus, and potassium were 450, 225, and 450 kg/ha, respectively. The chemical fertilizers were urea (N 46%), superphosphate (P 12%), and potassium chloride (K 60%). The organic fertilizer was a commercial organic fertilizer made primarily from sheep manure (purchased from Hebei Ningzhou Biotechnology Co., Ltd., Baoding, China), with nitrogen, phosphorus, and potassium contents of 14.0, 20.0, and 22.1 g/kg, respectively. The application rate of organic fertilizer was calculated based on the application rate of nitrogen fertilizer. Since organic fertilizer also contains phosphorus and potassium, the phosphorus and potassium content in the organic fertilizer should be deducted when applying chemical fertilizers. All organic fertilizer, 20% nitrogen fertilizer, 100% phosphorus fertilizer, and 30% potassium fertilizer were applied as base fertilizer by broadcasting. The remaining fertilizer was divided into six equal portions and applied to the soil on 10 May, 20 May, 27 May, 3 June, 10 June, and 16 June each year, respectively. Agronomic management practices followed local farmers’ customs (see Supplementary Materials for details). For this study, leaf (seventh true leaf) and fruit samples were collected on 13 June 2024 (BBCH 83). Five representative samples were collected from each plot, for a total of 15 samples per treatment. These samples were stored at −80 °C and −20 °C for subsequent determination of carbon and nitrogen metabolic activity and quality indicators. Cucumber yield was calculated by summing the total weight of cucumbers harvested every two days between 7 June 2024 (BBCH 81) and 18 July 2024 (BBCH 92).

### 2.3. Sampling and Measurements

#### 2.3.1. Nitrogen Assimilation

The amino acid (AA) content in cucumber leaves was determined according to the method of Ruiz and Romero [14]. The protein content was determined using the bicinchoninic acid (BCA) protein content determination kit (Qi Yi Biological Technology Co., Ltd., Shanghai, China). The principle is that under alkaline conditions, cysteine, cystine, tryptophan, etc., in protein can reduce Cu^2+^ to Cu^+^, and two molecules of BCA combine with Cu^+^ to form a purple complex with the strongest absorption peak at 562 nm. The enzyme activities of Glutamate synthase (GOGAT) and Glutamine synthetase (GS) were determined according to the Borlotti et al. method [15].

#### 2.3.2. Carbon Metabolism

Glucose, sucrose, and fructose contents were determined according to Araya et al. [16]. The activities of fructose-1,6-bisphosphate aldolase (FBA), sucrose phosphate synthase (SPS), and ribulose-1,5-bisphosphate carboxylase/oxygenase (Rubisco) were determined according to the reference kits (Qi Yi Biological Technology Co., Ltd., Shanghai, China).

#### 2.3.3. Stress Resistance

The determination of malondialdehyde (MDA) content was based on the description of Jiang et al. [17]. The basis for the determination of total antioxidant capacity (T-AOC) is that under acidic conditions, the ability of a substance to reduce Fe^3+^-tripyridine triazine (Fe^3+^-TPTZ) to produce blue Fe^2+^-TPTZ can reflect its total antioxidant capacity. For detailed operation procedures, please refer to the total antioxidant capacity determination kit (Qi Yi Biological Technology Co., Ltd., Shanghai, China).

#### 2.3.4. Fruit Quality

Cucumber fruit samples under different treatment conditions were collected at the maturity stage, including 5 biological replicates, and the vitamin C (AsA), soluble sugar (SS), and soluble solid (SSC) contents were determined as described by Wang et al. [2].

#### 2.3.5. Yield

Cucumbers were harvested starting on 7 June 2024 (the ripening period), and were harvested every two days until 18 July (the end of the cucumber life cycle). The total cucumber yield of each treatment group was recorded in t/ha.

### 2.4. Statistical Analysis

One-way analysis of variance (ANOVA) and Duncan’s multiple comparison test were performed using SPSS 24.0, with a significance level of *p* < 0.05. Data are presented as mean ± standard deviation. Pearson correlation analysis was performed using OriginPro 2021 software. Principal component analysis (PCA) and partial least squares discriminant analysis (PLS-DA) were performed using Metabo Analyst 6.0 (https://www.metaboanalyst.ca).

## 3. Results

### 3.1. Effects of OFS on Cucumber Yield

Table 2 shows that both chemical and organic fertilizers significantly increased cucumber yield. Compared with the NPK treatment (51.2 t/ha), organic fertilizer further increased cucumber yield. When the OFS ratio was 1/3 (2/3 NPK + 1/3 M), the cucumber yield reached 53.9 t/ha; when the OFS ratio was 2/3 (1/3 NPK + 2/3 M), the cucumber yield reached the highest value of 65.2 t/ha, which was 77.66% higher than the control treatment.

### 3.2. Effects of OFS on Cucumber Quality

**Figure 1:**
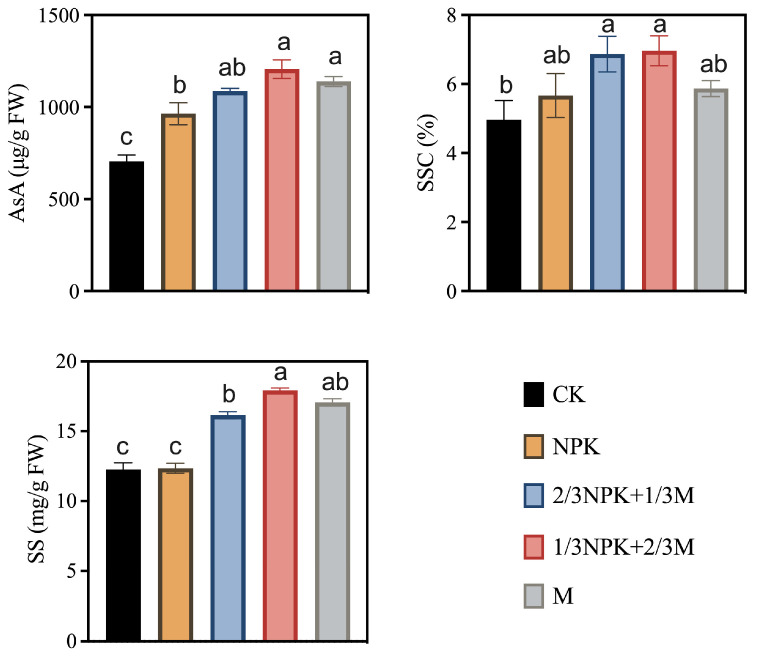
Effects of OFS on cucumber quality. ASA, vitamin C; SS, soluble sugar; SSC, soluble solids. Different lowercase letters indicate significant differences among treatments.

Figure 1 shows that both chemical and organic fertilizers improved cucumber fruit quality, but organic fertilizer was more effective than chemical. We also observed that with increasing OFS ratios, the AsA, SS, and SSC in cucumber fruits initially increased and then decreased. The optimal treatment was 1/3 NPK + 2/3 M. Under this treatment, the contents of AsA, SS, and SSC in cucumber fruits reached 1206.45 μg/g, 17.94 mg/g, and 6.97%, respectively, significantly higher than the CK treatment by 70.98%, 46.20%, and 40.27%.

### 3.3. Effects of OFS on Cucumber Stress Resistance

**Figure 2:**
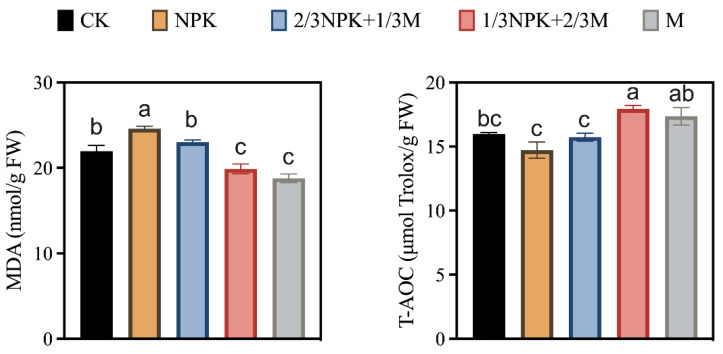
Effects of OFS on the antioxidant level of cucumber. MDA, malondialdehyde; T-AOC, total antioxidant capacity. Different lowercase letters indicate significant differences among treatments.

Figure 2 shows that applying organic fertilizer in the field trial helped improve the stress resistance of cucumbers. Compared with the CK treatment, the MDA content in the NPK treatment was significantly increased by 11.94%, and the MDA content gradually decreased with the increase in the OFS ratio. In the M treatment, the MDA content in leaves was 18.78 nmol/g, significantly lower than the control by 14.48%. Conversely to the trend of MDA content change, the total antioxidant capacity (T-AOC) in the NPK treatment was 14.72 μmol Trolox/g, and the T-AOC in leaves gradually increased with the increase in the OFS ratio. When the OFS ratio increased to 2/3, the T-AOC in leaves was 17.94 μmol Trolox/g, an increase of 12.28% compared with the CK treatment.

### 3.4. Effects of OFS on Cucumber Metabolites

**Figure 3:**
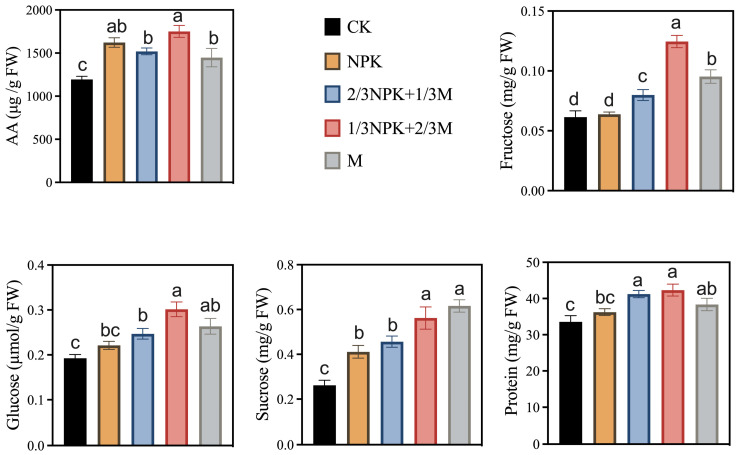
Effects of OFS on the accumulation of carbon and nitrogen metabolic assimilation products in cucumber. AA, amino acid. Different lowercase letters indicate significant differences among treatments.

Figure 3 shows that compared with the CK treatment, the NPK treatment significantly increased the contents of AA (1622.91 μg/g) and sucrose (0.41 mg/g) in cucumber leaves, with increases of 35.95% and 57.26%, respectively. The application of organic and inorganic fertilizers significantly improved the synthesis of carbon and nitrogen metabolites in cucumber. When 1/3 NPK + 2/3 M was applied, the contents of AA, fructose, glucose, sucrose, and protein in cucumber leaves were the highest, at 1751.55 μg/g, 0.12 mg/g, 0.30 μmol/g, 0.56 mg/g, and 42.33 mg/g, respectively, which were significantly increased by 46.72%, 102.57%, 56.70%, 114.90%, and 26.05% compared with the CK treatment.

### 3.5. Effects of OFS on the Activities of Metabolic Enzymes in Cucumber

**Figure 4:**
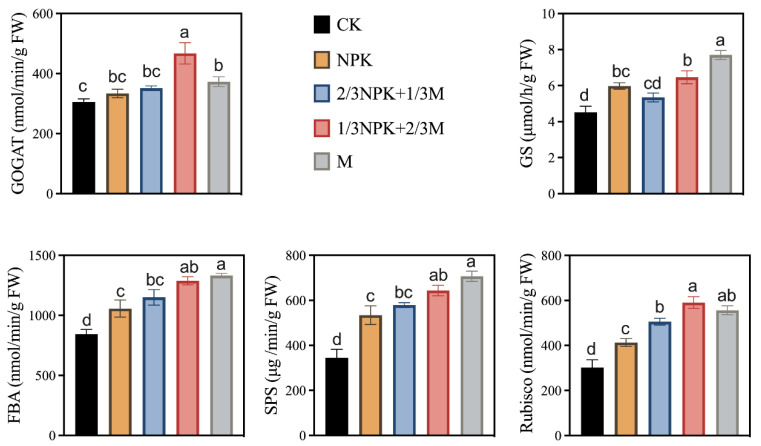
Effects of OFS on the activities of carbon and nitrogen metabolic enzymes in cucumber. GOGAT, glutamate synthetase; GS, glutamine synthetase; FBA, fructose-1,6-bisphosphate aldolase; SPS, sucrose phosphate synthase; Rubisco, ribulose-1,5-bisphosphate carboxylase/oxygenase. Different lowercase letters indicate significant differences among treatments.

Figure 4 shows that fertilization helps to increase the activities of carbon and nitrogen metabolism enzymes in cucumber leaves, but the responses of different enzymes to fertilization strategies show great differences. Compared with the CK treatment, the NPK treatment and the 2/3 NPK + 1/3 M treatment had relatively small effects on the activity of GOGAT. With the increase in the OFS ratio (1/3 NPK + 2/3 M), the GOGAT enzyme activity reached the maximum value (467.25 nmol/min/g). In addition, fertilization led to the increase in GS, FBA, SPS, and Rubisco enzyme activities, and the effect of OFS was better than the NPK treatment. Although the FBA and SPS enzyme activities were the highest in the M treatment, reaching 1287.86 nmol/min/g and 643.83 μg/min/g, respectively, there was no significant difference compared with the 1/3 NPK + 2/3M treatment (*p* > 0.05).

### 3.6. Pearson Correlation Analysis of Carbon and Nitrogen Metabolism Activities in Cucumber

**Figure 5:**
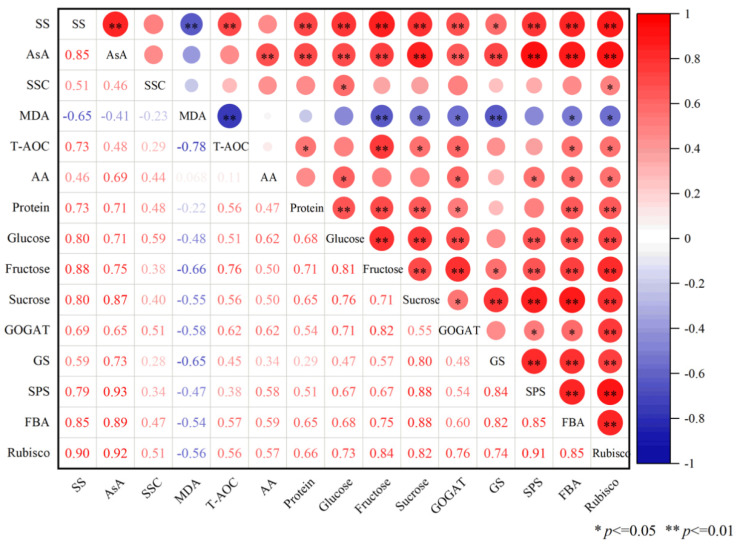
Pearson correlation analysis of carbon and nitrogen metabolic activities. SS, soluble sugar; AsA, vitamin C; SSC, soluble solids; MDA, malondialdehyde; T-AOC, total antioxidant capacity; AA, amino acid; GOGAT, glutamate synthetase; GS, glutamine synthetase; SPS, sucrose phosphate synthase; FBA, fructose-1,6-bisphosphate aldolase; Rubisco, ribulose-1,5-bisphosphate carboxylase/oxygenase. The numbers on the figure represent the correlation coefficient; red circles represent positive correlation, and blue circles represent negative correlation, which * represents *p* ≤ 0.05, and ** represents *p* ≤ 0.01.

The results of correlation analysis showed that carbon metabolism was positively correlated with nitrogen assimilation activity, while MDA was negatively correlated with almost all indicators (Figure 5). Among the quality indicators, SS, AsA, and SSC, only SS was highly significantly positively correlated with AsA (r = 0.85, *p* < 0.01). The T-AOC of leaves was correlated with SS (r = 0.73), protein (r = 0.56), fructose (r = 0.76), sucrose (r = 0.56), GOGAT (r = 0.62), FBA (r = 0.57), and Rubisco (r = 0.56), which were all significantly positively correlated. AA and protein were significantly positively correlated with GOGAT, glucose, FBA, and Rubisco. Glucose, fructose, and sucrose were significantly positively correlated with SPS, FBA, and Rubisco. GS and GOGAT were significantly positively correlated with SPS, FBA, and Rubisco.

### 3.7. The Overall Response of the Cucumber’s Metabolic Activities to OFS

**Figure 6:**
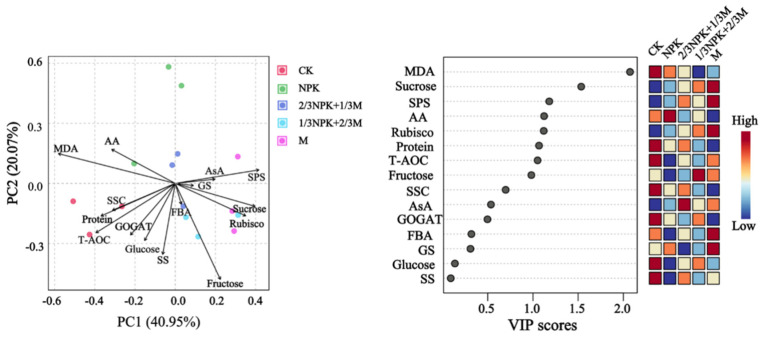
PCA and PLS-DA of carbon metabolism, nitrogen assimilation, and quality indicators. Relevant indicators are represented by arrows, and different treatments are represented by circles of different colors. MDA, malondialdehyde; SPS, sucrose phosphate synthase; AA, amino acid; Rubisco, ribulose-1,5-bisphosphate carboxylase/oxygenase; T-AOC, total antioxidant capacity; SSC, soluble solids; ASA, vitamin C; GOGAT, glutamate synthetase; FBA, fructose-1,6-bisphosphate aldolase; GS, glutamine synthetase; SS, soluble sugar.

PCA and PLS-DA were used to analyze leaf carbon metabolism and nitrogen assimilation, as well as fruit quality indicators, to clarify the response characteristics of cucumber to different proportions of OFS (Figure 6). The results of PCA showed that the PC1 and PC2 axes revealed 40.95% and 20.07% of the total variation, respectively. The points representing the CK treatment were mainly concentrated in the lower left part of the score graph. The application of inorganic fertilizer (M) to the soil moved it upward along the PC2 axis, while the application of organic fertilizer moved it to the right along the PC1 axis. At the same time, there were some intersections between the 1/3 NPK + 2/3 M treatment and the M treatment, indicating that the enzyme activity and metabolite content between the two treatments were similar. Furthermore, this study conducted PLS-DA analysis on the aforementioned data to explore the contribution (VIP) of each indicator to the classification. Cross-validation results showed that the model had R^2^ = 0.872 and Q^2^ = 0.766, indicating that the model is reliable and can be used for subsequent analysis. Based on the criteria of VIP > 1.5 and *p* < 0.05, MDA (VIP = 2.07) and sucrose (VIP = 1.53) were selected, indicating that these two indicators are important characteristics for distinguishing different combinations of organic and inorganic fertilizers.

## 4. Discussion

### 4.1. Effects of OFS on Cucumber Yield and Quality

In the critical stage of crop growth, if the nutrients required cannot be supplemented in time, the photosynthetic rate of crops will decrease, the transport of photosynthetic products to fruits will decrease, and the yield and quality will be reduced. Therefore, the availability of nutrients in the soil is considered to be an important factor in regulating crop production activities [18]. This study found that OFS significantly increased the activity of FBA, SPS, and Rubisco in cucumbers (Figure 4) as well as the content of AA, glucose, sucrose, and protein (Figure 3), which was significantly positively correlated with the improvement of SS and ASA in quality (Figure 1 and Figure 5). Compared with the 2/3 NPK + 1/3 M treatment, the cucumber yield and quality of the 1/3 NPK + 2/3 M treatment were the highest. Wang et al. confirmed that in the presence of organic fertilizer, reducing the amount of chemical fertilizer will not have an adverse effect on soil properties and cucumber yield, and organic fertilizer can increase the number of aerobic bacteria, Gram-negative bacteria, and saprophytic fungi in the soil, as well as the activity of soil urease, alkaline phosphatase, and sucrase, thereby ensuring the effective supply of soil nutrients [2]. Agbor et al. confirmed that applying organic fertilizer could increase cucumber yield by 105.45% compared to no fertilizer application and that there was a strong correlation between the total nitrogen, available phosphorus, and potassium content in the soil and cucumber yield [19]. In addition, we also noted that the yield of the 1/3 NPK + 2/3 M treatment was relatively higher than that of the M treatment, which is consistent with the research results of Mo-hamed et al.; that is, OFS has a higher cucumber yield than applying organic fertilizer or inorganic fertilizer alone and is beneficial to promoting the absorption and utilization of soil nutrients by plants [20]. In summary, these results indicate that OFS may improve the yield and quality of cucumbers by improving the absorption of soil nutrients by plants and promoting the synthesis of photosynthetic products and their translocation to fruits.

### 4.2. Effects of OFS on Metabolic Activities of Cucumber

Plant defensive metabolism is affected by fertilization activities [21]. Malondialdehyde is generally considered a biomarker of lipid peroxidation [22]. This study found that the application of NPK caused a significant increase of 11.94% in the MDA content of cucumber leaves, while the 1/3 NPK + 2/3 M and M treatments significantly reduced its content (Figure 2). Wang et al. demonstrated that organic fertilizer can provide plants with a balanced supply of nutrients, promote soil health, and help reduce oxidative stress damage and hydrogen peroxide production [23]. The results of the total antioxidant capacity test also confirmed that the application of chemical fertilizers will lead to a decrease in the T-AOC of cucumber leaves. After replacing 2/3 of the chemical fertilizers with organic fertilizers, the T-AOC increased significantly by 12.28%, indicating that OFS can better maintain crop yields and the sustainability of agricultural ecosystems.

Maintaining carbon and nitrogen assimilation is the basis for improving crop stress resistance without reducing yield [24]. Leaves rely on their complex internal biochemical photosynthesis mechanism to capture light energy and reduce carbon dioxide (CO_2_) and nitrate ions (NO_3_^−^) to carbohydrates and amino acids. Non-structural carbohydrates, as the main photosynthetic product of plants, can usually reflect the overall carbon supply status of plants [25]. This study found that the application of organic fertilizers (2/3 NPK + 1/3 M, 1/3 NPK + 2/3 M, and M) promoted the accumulation of fructose, glucose, sucrose, amino acids, and proteins in cucumber leaves, while the application of inorganic fertilizers alone only significantly increased the content of AA and sucrose (Figure 3). Glucose and sucrose, as the primary storage forms of photosynthetic products, provide energy sources and basic carbon skeletons for nitrogen assimilation but are easily limited by nitrogen availability. The OFS can gradually release nutrients, increase nitrogen absorption, and thus promote cucumber yield [26]. Carbon and nitrogen metabolites control the flow of carbon and nitrogen by regulating the activities of related enzymes and transporters, thereby responding to changes in the external environment and the source-sink relationship in the plant body [27]. The results of this study showed that both chemical and organic fertilizers helped to increase the activities of GOGAT, GS, FBA, SPS, and Rubisco enzymes in cucumber leaves. However, compared with NPK treatment, the 1/3 NPK + 2/3 M treatment significantly enhanced the activities of carbon and nitrogen metabolic enzymes; with increasing OFS ratio, the enhancement of most enzyme activities (such as FBA, SPS, and Rubisco) gradually weakened (Figure 4). The main protein in chloroplasts is Rubisco, which is involved in catalyzing the synthesis of triose phosphate from CO_2_ and ribulose-1,5-bisphosphate (RuBP). Triose phosphate is transported from chloroplasts to the cytoplasm and produces sucrose under the action of SPS. In fact, to achieve maximum CO_2_ assimilation, the nitrogen supply during leaf growth needs to be equivalent to the level required to maintain the potential protein synthesis rate [28]. The electrons transferred by photosynthetic electrons can reduce NO_3_^−^ to NH_3_ under the action of nitrate reduction and nitrite reductase and further convert them into amino acids through GS/GOGAT enzymes. GS and GOGAT are key enzymes for nitrogen assimilation. Studies have shown that reduced GS activity inhibits the synthesis and conversion of amino acids, thereby affecting the synthesis of proteins and all cellular components, while strains overexpressing GS help promote the accumulation of rice yield [29], which is consistent with the results of this study that fertilization promotes cucumber yield growth (Table 2).

### 4.3. Comprehensive Analysis of the Response of Cucumber Carbon-Nitrogen Metabolic Activities to OFS

Since carbon metabolism can provide carbon skeletons and energy for nitrogen assimilation, and nitrogen assimilation can provide enzymes and photosynthetic pigments for carbon metabolism, and both of which require common reducing power and energy, understanding the interaction between carbon and nitrogen metabolism can provide ideas for improving crop yield and quality [30]. Pearson correlation analysis showed that amino acids and proteins were significantly positively correlated with glucose, fructose, sucrose, SPS, FBA, and Rubisco (*p* < 0.05). Vicente et al. also confirmed through network analysis in a study on the effects of CO_2_, nitrogen, and temperature levels on wheat carbon and nitrogen metabolism that about 95% of carbon and nitrogen metabolism biochemical parameters and transcription levels from different treatment groups were significantly positively correlated, revealing that nitrogen absorption and assimilation are combined with photosynthesis, photorespiration, and respiration in carbon metabolism [31]. In addition, during the normal growth and development of plants, photosynthesis and other metabolic activities may also lead to the accumulation of hydrogen peroxide and membrane lipid peroxidation products in the plant [32]. The large amount of these substances is detrimental to plants and is mitigated by the antioxidant defence system. This is why we observed a significant negative correlation between MDA and most indicators. Carbon and nitrogen metabolites can regulate the activity of enzymes and transport proteins, thereby controlling carbon and nitrogen flux. A high level of carbon and nitrogen assimilation homeostasis is essential for enhancing plant resistance to adverse environments [33]. In addition, our results also showed that SS and AsA in cucumber quality indicators were significantly positively correlated with most carbon and nitrogen metabolic indicators. This indicates that OFS can enhance the carbon and nitrogen metabolism activity of cucumber leaves, promote the accumulation of their products, and provide sufficient substrates and energy for fruit formation and quality improvement. In order to further study its internal regulatory mechanism, we conducted PCA and PLS-DA analyses on the quality traits, carbon and nitrogen metabolic enzymes and products, and antioxidant levels of cucumber (Figure 6). The results showed that the application of chemical fertilizers and organic fertilizers would cause the metabolic activities of cucumbers to deviate upward and to the right along the PC2 and PC1 axes, respectively, and malondialdehyde and sucrose (VIP > 1.5, *p* < 0.05) were the two variables most significantly affected. Nitrogen is involved in the regulation of sugar metabolism in plants. Sucrose is an important source of carbon and energy for plants and is also a signal substance [27]. Studies have shown that appropriate nitrogen supply helps increase the sucrose content in grains, and exogenous application of 1% sucrose can alleviate the inhibition of high nitrogen supply on nitrogen metabolism activities, promote the accumulation of free amino acids, and promote the distribution of nitrogen to the aboveground parts [34,35].

## 5. Conclusions

**Figure 7:**
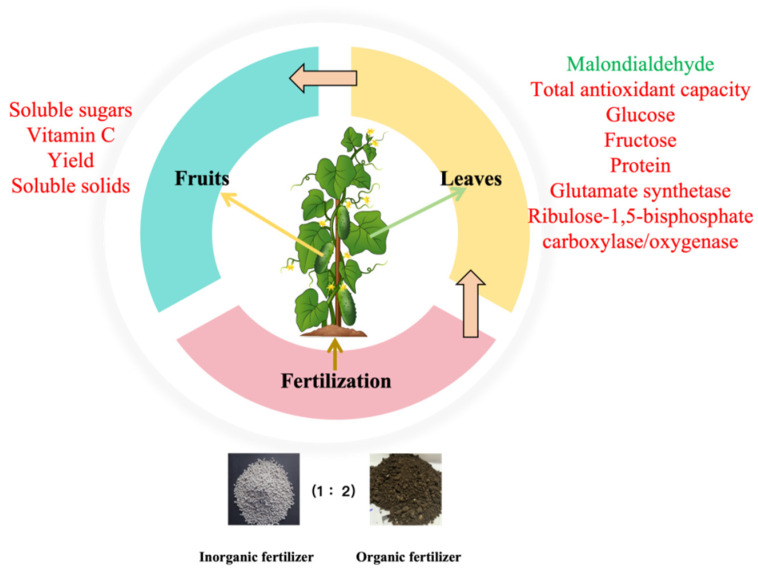
Schematic diagram illustrating how the OFS improves carbon and nitrogen metabolism and antioxidant capacity in cucumbers, thereby increasing their yield and quality. Red and green text in the figure represent increases and decreases, respectively.

Long-term application of chemical fertilizers may lead to soil degradation and productivity deterioration, while OFS can effectively reduce nutrient loss and achieve sustainable crop production. Compared with CK, NPK, 2/3NPK + 1/3M, and M treatments, the yield and quality indicators (ASA, soluble solids, and soluble sugars) of cucumbers in the 1/3NPK + 2/3M treatment were maximized. Antioxidant level analysis showed that the MDA content of cucumber leaves was significantly reduced and T-AOC was significantly increased under this treatment; carbon and nitrogen metabolism results showed that AA, fructose, glucose, sucrose, protein content, and GOGAT, GS, FBA, SPS, and Rubisco enzyme activities were significantly increased (Figure 7). Pearson correlation analysis of cucumber metabolic activities under different fertilization schemes confirmed that carbon metabolism and nitrogen assimilation showed a significant positive correlation and were closely related to the improvement of SS and AsA in cucumber quality indicators; PCA and PLS-DA results showed that 1/3NPK + 2/3M was significantly separated from other treatments, and sucrose and MDA obtained the largest variable weight values. Overall, the results of this study indicate that the 1/3 NPK + 2/3M fertilization strategy can significantly improve the carbon and nitrogen metabolic activities of cucumber, enhance its stress resistance, and promote yield and quality improvement. This strategy has important practical significance for alleviating soil obstacles caused by long-term continuous cropping and simultaneously improving crop resistance and fruit quality. However, this study still has some limitations, such as the lack of analysis on soil quality, heavy metal content, and soil microbial community structure, which need to be addressed in future work.

## Figures and Tables

**Table 1:** Fertilizer application rates under different organic–inorganic substitution patterns.

Treatment	Fertilizer (kg/hm^2^)			Organic Fertilizer (kg/hm^2^)		
	N	P_2_O_5_	K_2_O	N	P_2_O_5_	K_2_O
CK	0	0	0	0	0	0
NPK	450	225	450	0	0	0
2/3NPK + 1/3M	300	10.7	213.2	150	214.3	236.8
1/3NPK + 2/3M	150	0	0	300	428.6	473.6
M	0	0	0	450	642.9	710.4

Note: The nutrient contents of nitrogen, phosphorus, and potassium in sheep manure are 14.0, 20.0, and 22.1 g/kg, respectively. The nitrogen content in urea, the phosphorus content in superphosphate, and the potassium content in potassium chloride are 46%, 12%, and 60%, respectively.

**Table 2:** Effects of different OFS ratios on cucumber yield.

Treatment	Yield (t/ha)	Increase (%)
CK	36.7 ± 0.8 d	-
NPK	51.2 ± 0.5 c	39.51
2/3 NPK + 1/3 M	53.9 ± 0.1 b	46.87
1/3 NPK + 2/3 M	65.2 ± 0.7 a	77.66
M	55.6 ± 0.8 b	51.50

Note: Different lowercase letters indicate significant differences among treatments, *p* < 0.05. CK: no fertilizer; NPK: all inorganic fertilizer; 2/3 NPK + 1/3 M: 2/3 inorganic fertilizer plus 1/3 organic fertilizer; 1/3 NPK + 2/3 M: 1/3 inorganic fertilizer plus 2/3 organic fertilizer; M: all organic fertilizer.

## Data Availability

The original contributions presented in this study are included in the article/Supplementary Material. Further inquiries can be directed to the corresponding author.

## Supplementary Materials

The following supporting information can be downloaded at https://www.mdpi.com/article/10.3390/plants15142157/s1: Table S1: Total monthly rainfall and average temperature during the cucumber growing season from 2018 to 2024; Figure S1: Schematic diagram of field trials for different organic substitution models.
