# Supplementary material for: Organic–Inorganic Substitution Improves Cucumber Yield and Quality by Enhancing Carbon and Nitrogen Metabolism in Cucumbers
Source: Plants (Basel). 2026 Jul 13;15(14):2157. doi: 10.3390/plants15142157 (PMC13414767; doi:10.3390/plants15142157)
Supplement: Supplementary file 1 [file plants-15-02157-s001.zip › plants-4387774-supplementary.pdf]

# Supplementary Materials

**Local farmers' usual practices (2018-2024):**

May 1st: transplanting and watering cucumbers; May 7th: watering; May 9th: spraying pesticides to control aphids; May 18th: spraying pesticides to control caterpillars; May 19th: watering; May 24th: spraying pesticides to control whiteflies; June 1st: watering; June 13th: spraying pesticides to control aphids; June 20th: spraying pesticides; June 27th: watering; July 4th: watering and spraying pesticides.

**Table S1. Total monthly rainfall and average temperature during the cucumber growing season from 2018 to 2024**

| Year | Month | Monthly total rainfall (mm) | Average monthly temperature (℃) |
|---|---|---|---|
| 2018 | 5 | 27.1 | 23.05 |
| | 6 | 49.2 | 28.1 |
| | 7 | 240.2 | 29.9 |
| | 8 | 206.4 | 27 |
| 2019 | 5 | 22.8 | 23.7 |
| | 6 | 30.8 | 27.25 |
| | 7 | 133.4 | 29.05 |
| | 8 | 129 | 25.35 |
| 2020 | 5 | 43.3 | 22.1 |
| | 6 | 32 | 26.85 |
| | 7 | 60 | 29 |
| | 8 | 134.3 | 27.15 |
| 2021 | 5 | 5 | 19.45 |
| | 6 | 37.4 | 25.95 |
| | 7 | 338.7 | 26.65 |
| | 8 | 91.9 | 25 |
| 2022 | 5 | 25 | 21.9 |
| | 6 | 97 | 27.4 |
| | 7 | 176.1 | 26.65 |
| | 8 | 190.9 | 25.75 |
| 2023 | 5 | 28.9 | 23.3 |
| | 6 | 14.9 | 29 |
| | 7 | 255.9 | 30.8 |
| | 8 | 106.5 | 25.9 |
| 2024 | 5 | 33.9 | 22.3 |
| | 6 | 21.3 | 27.4 |
| | 7 | 136.9 | 28.5 |
| | 8 | 267.1 | 25.7 |

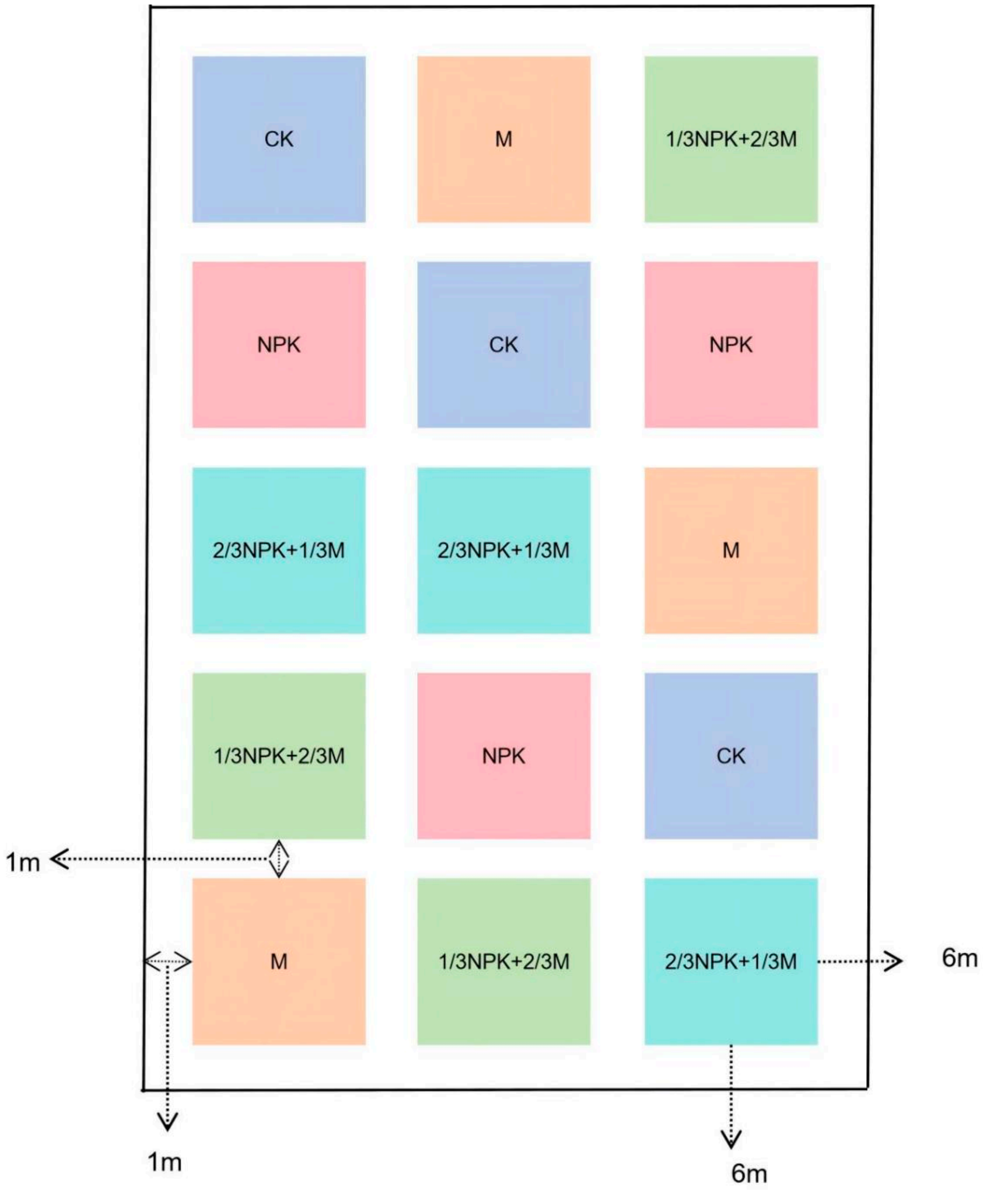


**Figure S1 Schematic diagram of field trials for different organic substitution models**

Note: CK: no fertilizer; NPK: all inorganic fertilizer; 2/3 NPK+1/3 M: 2/3 inorganic fertilizer plus 1/3 organic fertilizer; 1/3 NPK+2/3 M: 1/3 inorganic fertilizer plus 2/3 organic fertilizer; M: all organic fertilizer.
